# Long Non-Coding RNAs Involved in Progression of Non-Alcoholic Fatty Liver Disease to Steatohepatitis

**DOI:** 10.3390/cells10081883

**Published:** 2021-07-25

**Authors:** Biljana Atanasovska, Sander S. Rensen, Glenn Marsman, Ronit Shiri-Sverdlov, Sebo Withoff, Folkert Kuipers, Cisca Wijmenga, Bart van de Sluis, Jingyuan Fu

**Affiliations:** 1 Department of Pediatrics, University of Groningen, University Medical Center Groningen, 9713 GZ Groningen, The Netherlands; bibi_a_85@yahoo.com (B.A.); glennmarsman@hotmail.com (G.M.); f.kuipers@umcg.nl (F.K.); a.j.a.van.de.sluis@umcg.nl (B.v.d.S.); 2 Department of Genetics, University of Groningen, University Medical Center Groningen, 9713 GZ Groningen, The Netherlands; s.withoff@umcg.nl (S.W.); c.wijmenga@umcg.nl (C.W.); 3 Department of Surgery, Maastricht University and NUTRIM School for Nutrition and Translational Research in Metabolism, 6229 HX Maastricht, The Netherlands; s.rensen@maastrichtuniversity.nl; 4 Departments of Molecular Genetics, Molecular Cell Biology & Population Genetics, Nutrition & Toxicology Research (NUTRIM) Institutes of Maastricht, University of Maastricht, 6229 HX Maastricht, The Netherlands; r.sverdlov@maastrichtuniversity.nl; 5 Department of Laboratory Medicine, University of Groningen, University Medical Center Groningen, 9713 GZ Groningen, The Netherlands

**Keywords:** non-alcoholic fatty liver disease, functional genomics, long non-coding RNAs

## Abstract

Non-alcoholic fatty liver disease (NAFLD) is the most prevalent chronic liver disease and is characterized by different stages varying from benign fat accumulation to non-alcoholic steatohepatitis (NASH) that may progress to cirrhosis and liver cancer. In recent years, a regulatory role of long non-coding RNAs (lncRNAs) in NAFLD has emerged. Therefore, we aimed to characterize the still poorly understood lncRNA contribution to disease progression. Transcriptome analysis in 60 human liver samples with various degrees of NAFLD/NASH was combined with a functional genomics experiment in an in vitro model where we exposed HepG2 cells to free fatty acids (FFA) to induce steatosis, then stimulated them with tumor necrosis factor alpha (TNFα) to mimic inflammation. Bioinformatics analyses provided a functional prediction of novel lncRNAs. We further functionally characterized the involvement of one novel lncRNA in the nuclear-factor-kappa B (NF-κB) signaling pathway by its silencing in Hepatoma G2 (HepG2) cells. We identified 730 protein-coding genes and 18 lncRNAs that responded to FFA/TNFα and associated with human NASH phenotypes with consistent effect direction, with most being linked to inflammation. One novel intergenic lncRNA, designated lncTNF, was 20-fold up-regulated upon TNFα stimulation in HepG2 cells and positively correlated with lobular inflammation in human liver samples. Silencing lncTNF in HepG2 cells reduced NF-κB activity and suppressed expression of the NF-κB target genes *A20* and *NFKBIA*. The lncTNF we identified in the NF-κB signaling pathway may represent a novel target for controlling liver inflammation.

## 1. Introduction

Non-alcoholic fatty liver disease (NAFLD) is a complex disease that develops as a result of fat accumulation in the liver (fatty liver). During fat accumulation, lipid molecules in the form of triglycerides and cholesterol esters accumulate in the hepatocytes, a condition known as hepatic steatosis [1,2]. At the same time, other toxic lipid molecules such as free fatty acids (FFA) may cause hepatocyte injury (lipotoxicity) [3,4]. Liver inflammation can then be further aggravated by various mediators including endotoxins, lymphotoxin [5], adipokines, chemokines, and cytokines such as tumor necrosis factor alpha (TNFα) [6]. These molecules are secreted by different cells within the liver, including hepatocytes, and they can activate downstream pro-inflammatory signaling pathways such as the nuclear-factor-kappa B (NF-κB) signaling pathway [7]. Thereby, when combined with this inflammation, fatty liver will further result in non-alcoholic steatohepatitis (NASH) [8].

With development of high-throughput sequencing technologies such as RNA-sequencing (RNA-seq), transcriptome analysis has become a powerful tool to identify novel genes in NAFLD. It has also revealed that a large proportion of the human genome codes for long non-coding RNAs (lncRNAs) [9,10], which have been emerging as an important class of regulatory entities. Several lncRNAs have now been linked to liver metabolism and liver disease [11], including the recently reported liver-specific lncRNA, LIVAR, that is associated with hepatocyte viability and protection during NASH development [12]. However, characterizing lncRNA contributions to the complex progression of NAFLD has been a major challenge. Increasing evidence suggests that most lncRNAs are only active in a specific cell type and at certain development stages of diseases. Accordingly, despite the many lncRNAs that have been associated to NAFLD, little is known about their contribution to disease progression. Here, we present a framework that combines RNAseq experiments in 60 human liver samples with various degrees of NASH (Supplementary online 1) with a functional genomics experiment in an in vitro model in which we expose HepG2 cells to various stimuli to mimic NAFLD progression. Our in vitro model identified 4258 protein-coding genes and 109 lncRNAs that are likely involved in different stages of NALFD development, and 763 of these were also associated with human NASH phenotypes with the same effect direction. We further conducted a functional experiment that validated the involvement of one novel lncRNA, lncTNF, in the TNFα/NF-κB signaling pathway, and this may allow for development of novel therapeutics to reduce inflammation.

## 2. Materials and Methods

### 2.1. Cell Culture and Stimulation Experiments

HepG2 (ATCC) cells were kept at 37 °C and 5% CO_2_. The cells were cultured in Dulbecco’s Modified Eagle’s Medium (DMEM) containing Glutamax, supplemented with 1% (*v*/*v*) penicillin streptomycin (PS) and 10% (*v*/*v*) fetal calf serum (FCS). Before stimulation, HepG2 cells were cultured in a 6-well plate in DMEM until ~60–70% confluent. When confluent, the cells were starved for 24 h with starvation medium (DMEM+Glutamax and 1% PS, without 10% FCS). After 24 h, cells were stimulated for 24 h with media containing a combination of oleic acid and palmitic acid in a ratio of 2:1 (FFA concentration of 10 mM in 10% BSA, diluted 10 times in DMEM containing Glutamax and 1% PS, to final FFA concentration of 1mM) or 10% BSA medium (diluted 10 times in DMEM containing Glutamax and 1% PS). BSA medium was used as a control because FFAs are first bound to the BSA to increase the uptake of FFAs in the cells. After 24 h, the media were aspirated and either refreshed or changed with FFA+TNFα (1 mM FFA and 5 ng/mL TNFα) medium. RNA was isolated at different time points: 0 min (only stimulated with FFA or BSA for 24 h), 30 min, 3 h, and 5 h (BSA, FFA and FFA + TNFα). In total, we generated 33 samples: 11 conditions with triplicates per condition.

### 2.2. Oil Red O Staining

Lipid droplets were stained with ORO according to the manufacturer’s instructions (Biovision, Lipid (Oil Red O) Staining Kit). Nuclei were stained with hematoxylin. HepG2 cells were cultured in 6-well plates. When confluent, the cells were starved for 24 h by adding serum-free media to eliminate the effect of lipids from the serum. After starvation, cells were cultured in either FFA or BSA medium for 24 h. Next, the cells were washed twice with PBS, followed by fixation in 4% formaldehyde for at least 1 h. The cells were then washed twice with dH_2_O. After washing, 60% isopropanol was added for 5 min. Isopropanol was then aspirated, and cells were incubated for 10–15 min in ORO solution while shaking the plate gently. Cells were subsequently washed 5 times with dH_2_O to remove the excess ORO solution. After washing, cells were stained with hematoxylin for 30 s and washed thoroughly 5 times with dH_2_O to remove excess hematoxylin. Presence of lipid droplets was confirmed by light microscopy, followed by ORO extraction from the lipid droplets using 100% isopropanol. Optical density was measured by an ELISA plate reader at 500 nm.

### 2.3. RNA Sequencing of Hepatocyte Cell Lines

RNA was isolated using the Trizol method: 1 mL of Trizol was directly added to the cells in the 6-well plates and RNA was isolated according to the standard protocol. RNA concentration was measured with Nanodrop 1000 and RNA quality with LabChip GX. The average RNA integrity number (RIN) was 8. Sample preparation (*n* = 33) was done using the BiooScientific Nextflex kit, and paired-end sequencing was performed on the NextSeq500 sequencer. On average, ~40 million paired reads were produced per sample. All RNA-seq reads were aligned to the human genome (hg19) using STAR [13] and annotation of protein-coding genes and lncRNAs were based on the Gencode v7 catalog [10]. Rlog was normalized using the R package “DESeq2” [14]. The DESeq2 package was also used to analyze DE genes in the conditions versus controls. We performed the following DE analyses: FFA vs. BSA, FFA + TNFα vs. BSA, and FFA+TNFα vs. FFA. DE genes were considered significant at FDR < 0.1 and intersected with the human liver data (as described below). Significantly differentially expressed genes at FDR < 0.1 were used for pathway enrichment analysis using the DAVID database [15]. Spearman’s rank correlation coefficients were used to calculate the co-expressed genes (“guilt by association” approach). The analyses were performed in R version 3.4.1.

### 2.4. RNA Sequencing of Human Liver Samples

Liver biopsies from severely obese individuals (*n* = 60) were taken during bariatric surgery [16,17]. NAFLD severity was scored according to both the Brunt and Kleiner classifications (16 normal samples, 9 with NAFLD but no NASH, and 35 samples with different degrees of NASH). See Table S1 in the Supplementary Material for further relevant information on patient characteristics. Total RNA was extracted from frozen liver biopsy samples using RNeasy Mini Kit (Qiagen, Hilden, Germany), and RNA quality was assessed on an Agilent 2100 Bioanalyzer system (Agilent Technologies, Santa Clara, CA, USA). The average RIN was 7. cDNA libraries were prepared from total RNA, using SureSelectXT RNA Target Enrichment for Illumina Multiplexed Sequencing (Agilent Technologies), and were subjected to 100-bp paired-end sequencing on an Illumina HiSeq2500 Platform (Illumina, San Diego, CA, USA). Sequence reads from each sample were aligned to the reference human genome (UCSC hg19) in TopHat2 version 2.0.13 [18]. Reads aligned in TopHat2 were then assembled into a set of expressed transcripts based on the Gencode annotation (v7) [10], and Rlog was normalized using the DEseq2 package in R [14]. Expression data were corrected for age, age^2^, and gender. Corrected gene expression data were correlated with NASH phenotypes using the Spearman correlation test and corrected for multiple testing (FDR q-values). The same approach was used to calculate the co-expressed genes (“guilt by association” approach). The analyses were performed in R version 3.4.1.

### 2.5. Correlation of lncRNA Expression Profiles with NASH Phenotypes

The data were corrected for age, age^2^, and gender using a linear model that was run for all expressed genes. To determine the correlations between gene expression (*n* = 763 DE genes from the hepatocyte data) and NASH phenotypes, Spearman’s rank correlation coefficients were determined between gene expression values and the values of the measured traits, including NASH phenotypes. Permutation testing was performed for these correlations to estimate the FDR-corrected *p*-values. The same approach was used to calculate the co-expressed genes (“guilt by association” approach). For these genome-wide correlations, we used the FDR-corrected *p*-value.

### 2.6. Recombinant Adenovirus Ad5IκB and Viral Infection

The recombinant replication-deficient adenovirus Ad5IκB was generated as described previously [19]. It encodes hemagglutinin-tagged dominant negative human IκB (IκBα S32A/S36A) under the control of the CMV promoter. As a control, Cre vector was used, which is under the control of the CMV promoter as well as the IκBα vector. Ad5IkB was grown in HEK293 cells and purified by double cesium gradient and titered as described previously [19]. HepG2 were grown in 6-well culture plates in DMEM supplemented with 10% FBS and 1% P/S. At 70% confluency, HepG2 cells were infected with Ad5IκB at a multiplicity of infection of 50. After 48 h, virus-containing media was replaced with fresh media (controls) or media supplemented with TNFα (5 ng/mL) for 3 and 5 h. All conditions were run in triplicate. After the indicated time intervals, cells were washed with ice-cold PBS and RNA was isolated using the Tri-reagent as described below. cDNA was generated using the cDNA Master kit (Roche) according to the manufacturer’s instructions. We used qRT-PCR to measure the expression level of lncTNF. As a control gene, we used A20. Beta-actin was used as a reference gene. All primer sequences are shown in Supplementary Table S2.

### 2.7. lncTNF Knockdown

To knockdown lncTNF, we designed three shRNA cassettes for cloning into the lenti-viral pLKO TRC vector. The cassettes were specifically designed using the annotated lncTNF sequence. For this purpose, we used the siRNA selection program [20] and designed two shRNAs and one mock shRNAs. Cassette 1 was created by the annealing of shRNA1_FOR: CCGGTTGCCAGAGTCTAGGAGTTAACTCGAGTTAACTCCTAGACTCTGGCAATTTTTG and shRNA1_REV: AATTCAAAAATTGCCAGAGTCTAGGAGTTAACTCGAGTTAACTCCTAGACTCTGGCAA. Cassette 2 was created by the annealing of shRNA2_FOR: CCGGGAGCGTCATCCATTAATGCTTCTCGAGAAGCATTAATGGATGACGCTCTTTTTG and shRNA2_REV: AATTCAAAAAGAGCGTCATCCATTAATGCTTCTCGAGAAGCATTAATGGATGACGCTC. A mock shRNA hairpin was created based on oligos shRNA_mock_FOR: CCGGTTCTCCGAACGTGTCACGTGTCTCGAGACAGTGACACGTTCGGAGAATTTTTG and shRNA_mock_REV: AATTCAAAAATTCTCCGAACGTGTCACGTGTCTCGAGACACGTGACACGTTCGGAGAA.

Upon annealing of the oligos, the shRNAs were cloned into the pLKO TRC vector and lentiviral particles were produced as described previously [21]. Briefly, at 70% confluency, HEK293T cells were transfected (using PAI) with the vector containing lncRNA oligos together with the packaging vectors for lentiviral generation. After 48 h, virus media was collected and filtered to remove cell debris. The media was used to transduce target cells (HepG2). At 70% confluency, HepG2 cells were transduced with virus media (in 6-well plates using 3 wells per virus media). GFP-transduced cells were used as a control for the transduction. After 48 h, the media was changed with fresh DMEM medium without virus. The next day, puromycin selection was started (1 μg/mL medium) for up to 7 days, when stable cells were generated.

### 2.8. Cytoplasmic/Nuclear Fractionation

Subcellular fractionation was performed as previously described [12]. Briefly, full T75 flasks of HepG2 cells were harvested by trypsinization and centrifuged at 1000× *g* for 5 min. Cell pellets were resuspended in 300 µL of lysis buffer (140 mM NaCl, 1.5 mM MgCl2, 10 mM Tris-HCl pH8.0, 1mM DTT, 0.5% Nonidet P-40). Cells were incubated on ice for 5 min, followed by centrifugation for 5 min at 4 °C and 1000× *g*. The supernatant was collected in 2 mL Eppendorf tubes as the cytoplasmic fraction. Another 200 µL of lysis buffer was added to the remaining pellet, followed by centrifugation for 5 min at 4 °C and 1000× *g*. Supernatant was transferred to the cytoplasmic fraction. The nuclear fraction was washed once more with 200 µL of lysis buffer, followed by centrifugation for 5 min at 4 °C and 1000× *g*. The supernatant was discarded, and the remaining pellet was used as nuclear fraction. RNA was isolated, and samples were used for qPCR analysis.

### 2.9. RNA Isolation, cDNA Generation, and qRT-PCR Experiments

RNA was isolated using TRIzol reagent as described previously [12]. Isopropanol-precipitated and ethanol (70%)-washed RNA pellets were dissolved in RNase/DNase free water. cDNA was generated from 1 μg of RNA using the Transcriptor Universal cDNA Master kit (Roche) according to the manufacturer’s instructions. Gene expression was analyzed by qRT-PCR in an end volume of 10 µL with 5 µL SYBR Green, 2 µL cDNA template (20 ng), 2 µL RNAse/DNase free water (MQ), and 0.5 µL and 6 µM of forward and reverse primers, respectively. The following program was used: 50 °C/2 min, 95 °C/10 min, and 40 cycles with 95 °C/15 s and 60 °C /1 min. The plate was run on a QuantStudio 7 Flex Real-Time PCR System (Applied Biosystems/ ThermoFisher Scientific, Waltham, MA, USA), and the data were analyzed using the standard curve method on QuantStudio Real-Time PCR software. Primer sequences are shown in Supplementary Table S2.

### 2.10. Dual Luciferase Reporter Assay for Measurement of NF-κB Activity

The dual luciferase assay was used to explore the activity of NF-κB transcription factor in HepG2 cells expressing shRNA1/ shRNA2/ mock vector upon TNFα stimulation. For this purpose, we used a NF-κB-responsive luciferase reporter plasmid containing two canonical NF-κB sites [22]. Renilla luciferase pRL-SV40 vector was used to normalize and reduce differences in transfection efficiencies and subsequent variations in these experiments. Cells were seeded in 6-well plates in triplicate for each condition. After attachment at 60–70% confluency, cells were co-transfected with 1900 ng of the 2 κB-luc construct, 50 ng pRL-SV40/Renilla vector, and 50 ng empty vector using Lipofectamine 3000 (Invitrogen, Carlsbad, CA, USA). Transfection was performed according to the manufacturer’s protocol using 7.5 μl Lipofectamine 3000 reagent. TNFα (10 ng/mL) was added into the wells of the stimulation group 48 h later. After incubation of 6, 12, and 24 h with cell culture media containing TNFα, cells were lysed in passive lysis buffer (Promega, Madison, WI, USA). Firefly and Renilla luciferase signals were measured by the Dual-Luciferase^®^ Reporter Assay System (Promega) in a Synergy H4 Hybrid Microplate Reader (BioTek, Winooski, VT, USA). Relative luciferase activity (Luc), calculated by the ratio of Firefly and Renilla luciferase signals, was used to monitor NF-κB activity in lncTNF knock-down cells vs. controls.

## 3. Results

### 3.1. Identification of Differentially Expressed Genes and lncRNAs in Stimulated Conditions

**Figure 1:**
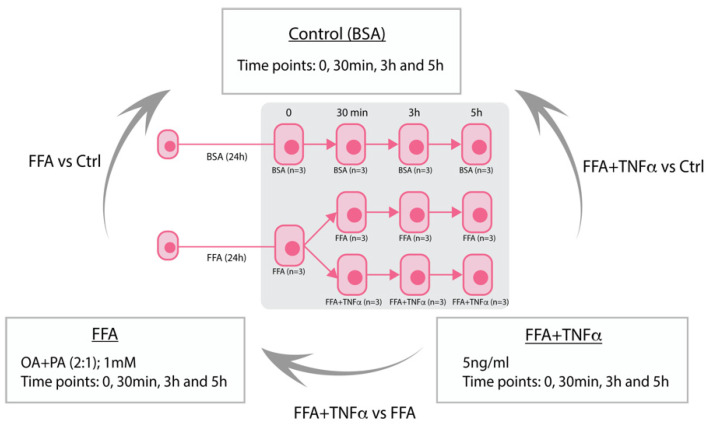
Study overview of HepG2 stimulation. Stimulation of HepG2 hepatocytes with free fatty acids (FFA) to mimic liver steatosis and tumor necrosis factor alpha (TNFα) to mimic liver inflammation in NASH. BSA (Bovine Serum Albumin).

HepG2 cells were exposed to free fatty acids (FFA) (unexposed, 30 min, 3 h, and 5 h) or FFA+TNFα stimulation (30 min, 3 h, and 5 h) versus bovine serum albumin (BSA) control conditions (unexposed, 30 min, 3 h, and 5 h) (Figure 1) with three biological replicates performed for each condition. Before performing RNA sequencing, FFA stimulation was confirmed by performing Oil Red O (ORO) staining on HepG2 cells in an extra 6-well plate. TNFα stimulation was confirmed by qRT-PCR analysis assessing the gene expression of two well-established responsive genes downstream from NF-κB—A20 and IκBα (Figure S1A,B). Presence of lipid droplets was confirmed by light microscopy (Figure S1C,D), followed by ORO extraction from the lipid droplets using 100% isopropanol. Optical density was measured by an ELISA plate reader at 500 nm (Figure S1E). RNAseq profiling generated expression of 24,701 protein-coding genes and 6799 lncRNA transcripts in 33 samples covering 11 conditions, with an average of 40 million reads generated per sample (Figure S2A). Principal-component analysis (PCA) across all samples showed that HepG2 cells expressed very distinct transcriptomes under different conditions (Figure S2B). PCA on the 24,701 mRNAs encoding protein-coding genes and the 6799 lncRNAs showed consistently similar patterns (Figure S2C,D, respectively).

**Figure 2:**
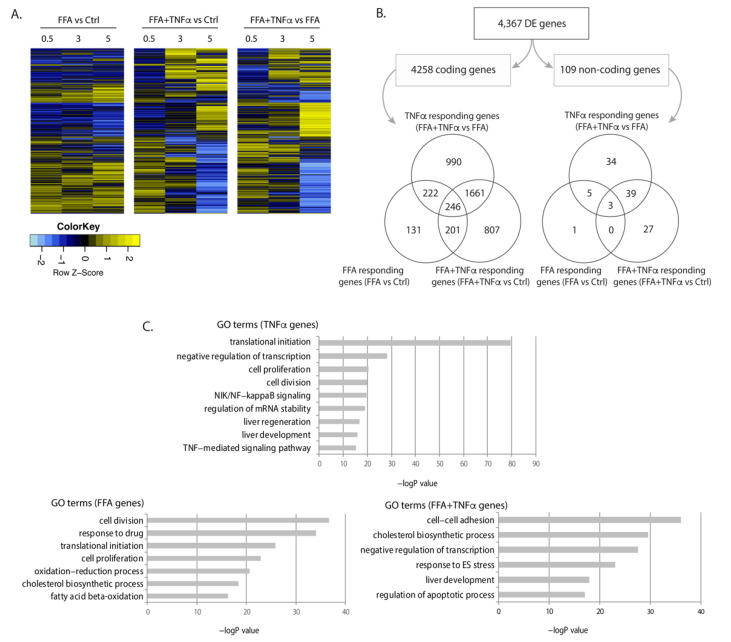
Differentially expressed genes upon FFA and TNFα stimulation. (**A**) Heatmap of differentially expressed (DE) genes in each condition at FDR < 0.1. Fold change of gene expression Z-scores is shown. (**B**) Number and Venn diagrams of DE genes at FDR < 0.1 in each condition for coding (left) and non-coding genes (right). (**C**) Pathway analysis for DE coding genes including gene ontology (GO) terms and false discovery rate (FDR) for each condition.

We conducted differential expression (DE) analysis: (1) between FFA and BSA conditions to identify genes that responded to FFA exposure, indicative of biological processes during fat accumulation; (2) between FFA and FFA+TNFα conditions to identify genes that responded to TNFα stimulation, indicative of biological processes occurring during liver inflammation; and (3) between FFA+TNFα and BSA controls to identify the accumulated effects of FFA and TNFα on gene expression. This identified 4367 DE genes (4258 mRNAs and 109 lncRNAs) at a false discovery rate (FDR) < 0.1 (Figure 2A,B, online data 1), with most genes being specific to a certain condition while some were shared between conditions. Consistent with the idea that excessive FFA-uptake by the cells is an initial step of NAFLD progression, genes differentially expressed upon FFA exposure were mostly those shared with other conditions. However, our study also revealed a large number of mRNAs and lncRNAs that only responded to TNFα or FFA+TNFα conditions, e.g., 990 mRNAs and 34 lncRNAs were only differentially expressed upon TNFα exposure, while 807 mRNAs and 27 lncRNAs were only differential expressed in the FFA+TNFα stimulated cells (Figure 2B).

Gene ontology (GO) term analyses of the regulated genes in each condition revealed that these regulated RNA transcriptomes clearly reflect condition-specific metabolic responses. As shown in Figure 2C, FFA-associated DE genes were enriched in cell division, oxidation-reduction processes, cell proliferation, and lipid metabolism pathways, while TNFα-associated DE genes were enriched in translation and transcription pathways, NF-κB signaling, Wnt signaling, and liver development. The combination of TNFα and FFA showed enrichment of genes involved in cholesterol biosynthesis, ER stress, liver development, and apoptosis. These results suggest that both protein-coding genes and lncRNAs may drive the hepatocyte response to FFA and TNFα exposure (online data 2).

### 3.2. Association of Differentially Expressed Genes with Human NASH

To determine which DE genes in the treated HepG2 cells could play a role in human NAFLD and NASH development, we conducted RNAseq experiments on 60 human liver samples with different degrees of NASH. For this purpose, expression values of the 4367 DE genes that showed a significant response in our stimulated HepG2 cells were extracted from the human liver RNAseq dataset and correlated with NASH phenotypes. Spearman correlation analysis revealed 763 DE genes that showed association with NASH phenotypes at FDR < 0.1 (online data 3) and the same effect direction in both datasets. This gene set included 730 protein-coding mRNA genes, 18 lncRNA genes, and 15 pseudogenes or processed transcripts. Some of these genes have previously been linked to NAFLD. For example, we observed that genes involved in lipid and FFA metabolism, such as *APOC1*, *APOA2*, *PPARA*, and *FADS2*, were down-regulated in both treated HepG2 cells and NAFLD livers (Figure S3A–D). *PPARA* also plays a role in liver inflammation [23]. Many inflammatory genes were upregulated in both datasets, including *IL8*, *CCL20*, *TNFAIP3*, and *TNFAIP8* (Figure S3E–H). Furthermore, *SOD2*, a gene that protects cells from superoxide radicals, was upregulated in cells upon FFA- and TNFα-exposure (Figure S3I) as well as in NASH livers. All these genes are known to play a role in NAFLD development [24,25,26]. Our results thus confirm that combining data from human liver samples and in vitro HepG2 experiments is a powerful and systematic approach to identifying genes involved in NAFLD and NASH progression. In human liver samples only, we totally detected significant association between hepatic expression of 3960 unique genes with NAFLD phenotypes at FDR < 0.1 (Figure S4A), including 854 lncRNAs and 3106 protein-coding and other genes. Negatively associated genes were enriched in oxidation-reduction pathways, the fatty-acid β-oxidation process, and various metabolic pathways (Figure S4B). Positively associated genes were enriched in inflammatory response pathways, signal transduction, response to lipopolysaccharides, and apoptotic processes, among the most significant pathways (Figure S4C).

**Figure 3:**
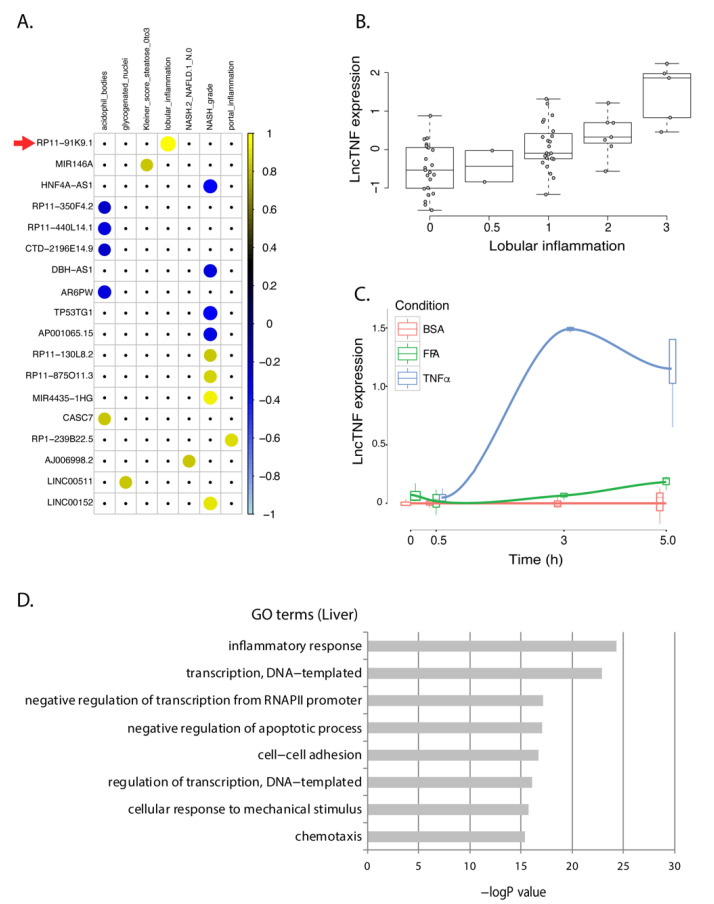
lncTNF expression in FFA- and TNFα-exposed HepG2 cells and correlation with NASH phenotypes in human livers. (**A**) Correlation plot representing the correlation coefficients (Spearman) between 18 NASH-associated lncRNAs that showed the same direction of dysregulation in the HepG2 model of NASH. Positive correlations are shown in yellow. Negative correlations are shown in blue. LncTNF, corresponding to RP11-91KP.1, is marked with a red arrow. Rows represent genes and columns represent NASH phenotypes. (**B**) Correlation between normalized lncTNF expression (*y*-axis) and NASH grade (*x*-axis) in human liver samples. (**C**) Normalized lncTNF expression (*y*-axis) in HepG2 cells upon exposure to free fatty acids (FFA, green line), tumor necrosis factor alpha (TNFα, blue line), or control conditions (BSA: bovine serum albumin, red line). Mean values for three replicates are shown. *x*-axis represents exposure time in hours. (**D**) Gene ontology (GO) terms (*y*-axis) and FDR corrected *p*-value (*x*-axis) as defined by DAVID. Genes co-expressed with lncTNF from the human liver data (FDR < 0.05) were used as input for this analysis.

Outside the established role of some protein-coding genes, knowledge of the involvement of lncRNAs in NAFLD progression in the liver is very limited. We identified 18 lncRNAs that showed consistent expression patterns in HepG2 cells and the human liver dataset. These lncRNAs mainly showed a response to TNFα-stimulation in HepG2 (Figure S5) and were associated with inflammation-related liver phenotypes (Figure 3A). None of their functions have been characterized before. Therefore, we used the GeneNetwork tool [27] to predict their functions (online data 4) and found that most showed co-expression with genes involved in interleukin and cytokine signaling. Furthermore, some other reaction pathways were also very interesting. For instance, our data have suggested NAFLD-associated lncRNAs might be involved in the regulation of endogenous sterols pathways. Evidence has suggested that sterol regulatory element-binding protein 1c (SREBP1c) is a critical regulator governing lipid homeostasis in the liver and its neddylation is a potential therapeutic target for NAFLD [28].

### 3.3. lncTNF: A Novel lncRNA Involved in Liver Inflammation

An intergenic lncRNA gene located on chromosome 3q26.32, annotated as RP11-91K9.1, showed the strongest response to TNFα stimulation in HepG2 cells. This lncRNA was positively associated to lobular inflammation (r = 0.58; *p* = 9.7 × 10^−7^) in human liver samples (Figure 3B). The baseline expression of this lncRNA in hepatocytes was low but showed a 20-fold increase upon TNFα stimulation after 3 h (Figure 3C). Therefore, we named it lncTNF because it may play a role in liver inflammation. Co-expression network analysis between lncTNF and protein-coding genes in human liver samples indicate that it is involved in inflammatory pathways, transcription processes, and negative regulation of apoptosis (Figure 3D; online data 5), which is consistent with GeneNetwork predictions (online data 4). These results suggest that lncTNF may be involved in liver inflammation and may contribute to NAFLD progression.

**Figure 4:**
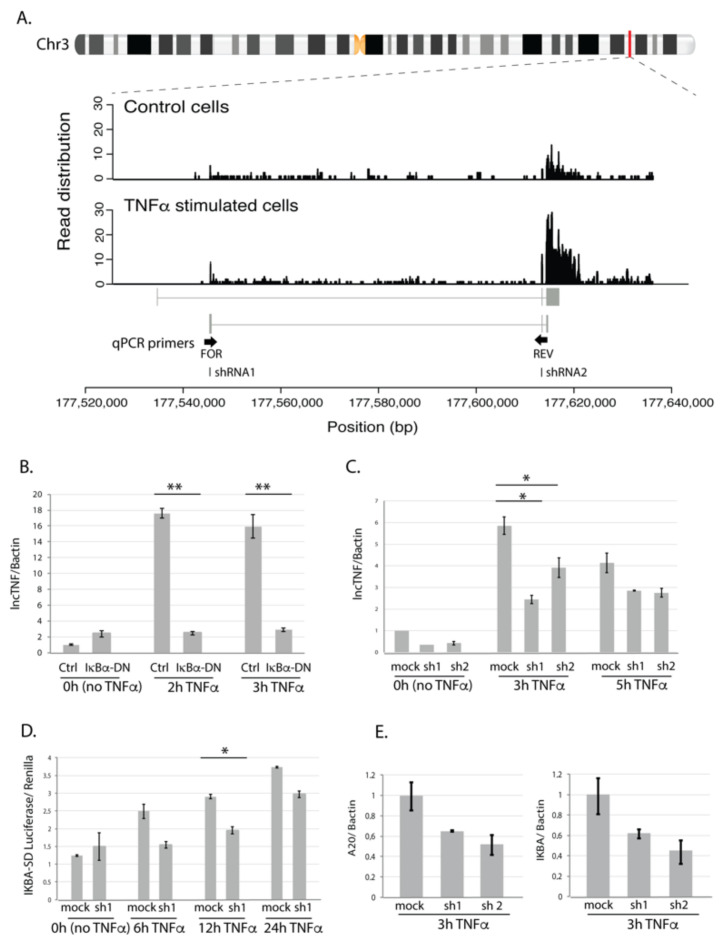
LncTNF structure and function. (**A**) Chromosomal location of lncTNF and read distribution based on the RNA sequencing in control (BSA-treated, all time points) and TNFα-stimulated cells (all time points). qPCR primers are located in exon 1 (forward primer) and exon 2 (reverse primer). shRNAs used for knock-down experiments are located in exon 1 (shRNA1) and exon 2 (shRNA2). (**B**) Gene expression of lncTNF relative to the Bactin (*y*-axis) measured by qPCR in HepG2 cells transduced with adenovirus containing IκBα dominant negative construct (Ad5IκB; IκBα-DN) and cells transfected with Cre adenovirus used as control (*x*-axis). Three time points were analyzed (0 h or no stimulation, 3 h, and 5 h of TNFα stimulation). (**C**) Gene expression of lncTNF relative to Bactin (*y*-axis) upon lncTNF knock-down using two different shRNAs (shRNA1 and 2) and three time points (*x*-axis). Scrambled shRNA sequence was used as control (mock). (**D**) Activity of NF-κB measured by luciferase/renilla ratio (*y*-axis) in lncTNF-KD cells (shRNA1) compared to mock control cells (*x*-axis). Transfected cells were stimulated with TNFα for 6, 12, and 24 h or not stimulated (0 h). (**E**) Gene expression level of A20 and IKBA relative to Bactin (*y*-axis) in lncTNF-KD cells and mock control cells upon 3 h TNFα stimulation. Values represent mean ± SEM, * = *p* ≤ 0.05, ** = *p* < 0.01.

When comparing lncTNF expression across 30 different tissue types from public datasets, we found that lncTNF showed the highest expression in the liver (Figure S6A). When comparing lncTNF expression in 66 cell types, we observed low expression of lncTNF in HepG2 cells (which is in line with our non-stimulated HepG2 cells) and high expression in epithelial cell-lines derived from the mammary gland and pancreas (Figure S6B). LncTNF has two possible isoforms annotated in GENCODE. However, our RNAseq data supported abundant expression of the second transcript isoform, very likely with a longer exon 3 (Figure 4A). We further conducted a quantitative real-time PCR (qRT-PCR) analysis to confirm the response of lncTNF to TNFα stimulation and assessed its expression every 30 min after stimulation. The data show that lncTNF up-regulation started at 1.5 h and saw a 25-fold increase at 2 h (Figure S7A). This response was specific to hepatocytes, as the expression of lncTNF did not show a response in human embryonic kidney HEK293T cells (Figure S7B) and only a modest response in human monocytic THP1 cells (Figure S7B). Interestingly, we found that lncTNF also responded to interleukin 1β (IL1β), a pro-inflammatory stimulus (Figure S7C). Both TNFα and IL1β activate the NF-κB pathway, suggesting a role for lncTNF in liver inflammation through NF-κB signaling.

### 3.4. lncTNF Is Activated by NF-κB

To assess if the expression of lncTNF is activated by NF-κB, we transduced HepG2 cells with adenovirus containing an IκBα-dominant negative construct (Ad5IκB) and used expression of A20 as a control (Figure S8). IκBα inhibits NF-κB by masking the nuclear localization signals of NF-κB proteins, thereby keeping them in an inactive state in the cytoplasm. While TNFα-stimulation increased the expression of lncTNF, the TNFα-induced expression of lncTNF was abolished in cells transduced with Ad5IκB (Figure 4B). This suggests that the expression of lncTNF might be controlled by NF-κB. Consistent with this, we identified a putative NF-κB binding site in the vicinity of the lncTNF promoter, further suggesting that the expression of lncTNF is directly regulated by NF-κB.

To investigate the role of lncTNF in inflammation, we stably silenced the expression of lncTNF using the pLKO-TRC lentiviral system and two short hairpin RNAs (shRNAs) targeting different regions of lncTNF (Figure 4A). In non-stimulated and TNFα-stimulated cells, the expression of lncTNF was reduced by 40–50% upon shRNA expression (Figure 4C). We assessed the effect of lncTNF on global NF-κB activity using a reporter assay to measure NF-κB activity in lncTNF-KD cells and control cells. The cells were transfected with an NF-κB-reporter vector and stimulated with TNFα. The activity of NF-κB measured by the luciferase/renilla ratio was lower in lncTNF-KD cells compared to that of control cells, showing borderline significance at 12 h (*p* = 0.05) upon TNFα stimulation (Figure 4D). Next, we determined the expression of several NF-κB target genes and observed that the gene-expression-levels of *TNFAIP3* (*A20*) and *NFKBIA* (*IκBα*) were reduced in lncTNF-silenced cells (Figure 4E). This was seen when cells were stimulated with TNFα, but not under basal conditions without stimulation. We further found that lncTNF expression was much higher in the cytoplasm compared to that in the nucleus of HepG2 cells (Figure S9). Together, these results suggest that lncTNF acts in the cytoplasm to control the activity of NF-κB.

## 4. Discussion

Hepatocytes are the most abundant liver cell type and are strongly affected during NASH development. NASH phenotypes such as steatosis and ballooning occur within hepatocytes [29,30,31]. Stress signals such as lipotoxicity, oxidative stress, ER stress, and inflammation can additionally affect hepatocyte function [30,32,33,34,35,36]. In this study, we used the human hepatocyte cell line HepG2 as a model for NASH and treated these cells with FFA to induce steatosis, followed by treatment with TNFα to mimic the inflammatory processes within the liver. Despite several limitations of cancer-derived HepG2 cell lines, it is still a commonly used in vitro cellular model to study liver disease due to practical reason. This approach identified genes involved in different pathways known to be involved in NAFLD pathogenesis, including redox processes, lipid metabolism, ER stress, and NF-κB signaling [37], which confirmed the validity of our approach. We also detected 18 lncRNAs with unknown function that showed differential expression between cells under the various conditions employed and in the livers of NAFLD patients with different severity of disease. Notably, human liver tissues and RNA samples had been frozen at -80 °C for several years before the RNAseq experiment. We cannot exclude the impact of long-term storage of samples on gene expression.

One of the most promising lncRNA candidates, RP11-91K9.1 (lncTNF), was shown to be upregulated in HepG2 cells after stimulation with the pro-inflammatory cytokines TNFα and IL1β. LncTNF also positively correlated with inflammation in the livers of NASH patients. In line with our data, another study reported lncTNF upregulation upon stimulation with pro-inflammatory cytokine IL1α and platelet-derived growth factor in smooth muscle cells [38]. Both TNFα and IL1β activate the NF-κB signaling pathway, one of the main signaling pathways linked to liver inflammation [7,39]. Rapid activation of NF-κB is critical for host defense against various classes of pathogens. After activation, NF-κB induces the expression of a broad set of genes involved in processes such as proliferation, cell survival, and differentiation, as well as genes encoding proinflammatory cytokines that control immune and inflammatory responses [40]. Our data indicate that lncTNF is an NF-κB target gene, as shown by the strong inhibition of lncTNF expression after NF-κB inhibition by overexpressing IκBα-SD. Transcription factor binding sites for NF-κB are present near the transcription start site of lncTNF, suggesting direct regulation by NF-κB. However, the locus also contains transcription motifs for other transcription factors that are NF-κB target genes, including c-Jun, CEBPB, p300, FOXA1, and FOXA2. The closest (-9nt from the transcription start site) is CEBPB, a transcription factor that regulates genes involved in immune and inflammatory responses and has a promitotic effect on many cell types such as hepatocytes and adipocytes [41]. Therefore, the expression of lncTNF may be directly and/or indirectly regulated by NF-κB.

NF-κB activation occurs in the cytoplasm of cells. Canonical activation of NF-κB by TNFα leads to recruitment of protein complexes that mediate signal-specific activation of IKK (I kappa B kinase) [42]. Upon activation, IKK phosphorylates IκB proteins (IκBα, IκBβ, and IκBε), which triggers their K48-linked polyubiquitination and subsequent degradation by the proteasome. The three IκB proteins are regulated by different NF-κB mechanisms, and IκBα is degraded most rapidly in response to inflammatory stimuli compared to the speed of degradation of the other IκB proteins [43]. These events allow translocation of NF-κB into the nucleus and activation of NF-κB target genes. In this way, genes that encode IκBα and A20 are activated. After protein synthesis, IκBα binds to nuclear NF-κB complexes and inhibits their function by translocating NF-κB back into the cytosol [44]. The ubiquitin-editing enzyme A20 also down-regulates NF-κB, providing an additional negative feedback loop [45]. A20 removes polyubiquitin chains from RIP1 (one of the protein complexes that mediates signal-specific activation of IKK) and IKKγ, which leads to destabilization of the IKK-activation complex. This process shuts down the inflammatory response. Our lncTNF-knockdown experiment resulted in lower NF-κB activity and down-regulation of A20 and IκBα gene expression. Based on our data, a plausible hypothesis is that lncTNF regulates polyubiquitination processes, as also suggested by our pathway analysis, and acts as a positive feedback loop to regulate the inflammatory NF-κB response. However, this hypothesis needs further functional validation.

## 5. Conclusions

In summary, we present a functional genomics approach that systematically compared a HepG2-based in vitro model for NASH progression with human liver samples with different degrees of NASH. We identified 763 genes, 18 of which encode lncRNAs, which likely contribute to NASH progression. One of these, which we designated lncTNF, may have a role in NF-κB signaling and regulation of inflammation in hepatocytes and liver. Since treatments options for NASH are limited and the mechanisms by which NAFLD progresses to NASH are not well understood, the discovery of lncRNAs associated with NASH may lead to a better understanding of NAFLD progression and offer novel targets for treatment.

## Data Availability

The online datasets are available from https://github.com/JingyuanFu/Summary-statistics-of-NASH-lncRNA; The RNAseq data presented in this study are available from the European Nucleotide Archive (ENA) database via the access number PRJEB46457.

## Supplementary Materials

The following are available online at https://www.mdpi.com/article/10.3390/cells10081883/s1, Table S1: Plasma and clinical parameters of the MORE study population, Table S2: Primer sequences; Figure S1: Induction of inflammation and steatosis in HepG2 cells, Figure S2: RNA sequencing reads distribution and PC analysis, Figure S3: Normalized expression levels of known NASH dis-regulated genes in the HepG2 dataset. Figure S4: Genes correlated with NALFD phenotypes in human liver samples, Figure S5: lncRNAs that showed consistent expression patterns in challenged HepG2 cells and in human liver datasets. Figure S6: Expression levels of lncTNF in publicly available RNAseq datasets (European Nucleotide Archive). Figure S7: qRT-PCR expression of lncTNF upon different cytokine treatments and among different cell lines. Figure S8. qRT-PCR measurement of A20 gene expression relative to B-actin gene (*y*-axis) in HepG2 cells treated with Cre adenovirus (Ctrl) and Ad5IκB (IκBα-DN) adenovirus media (*x*-axis). Figure S9: Cellular localization of lncTNF in HepG2 cells.
