# Supplementary material for: Long Non-Coding RNAs Involved in Progression of Non-Alcoholic Fatty Liver Disease to Steatohepatitis
Source: Cells. 2021 Jul 25;10(8):1883. doi: 10.3390/cells10081883 (PMC8394311; doi:10.3390/cells10081883)

Supplementary Material

Atanasovska et al.

Long Non-Coding RNAs Involved in Progression of non-Alcoholic Fatty Liver Disease to Steatohepatitis

Table of Contents

Table S1. Plasma and clinical parameters of the MORE study population..................... 2

Table S2. Primer sequences.. 3

Figure S1. Induction of inflammation and steatosis in HepG2 cells................................ 4

Figure S2. RNA sequencing reads distribution and PC analysis..................................... 5

Figure S3. Normalized expression levels of known NASH dis-regulated genes in the HepG2 dataset.. 6

Figure S4. Genes correlated with NAFLD phenotypes in human liver samples........... 7

Figure S5. 18 lncRNAs which showed consistent expression patterns in challenged HepG2 cells and in human liver datasets.. 8

Figure S6. Exprssion levels of lncTNF in publicly available RNAseq datasets (European Nucleotide Archive).. 9

Figure S7. qRT-PCR expression of lncTNF upon different cytokine treatments and among different cell lines.. 10

Figure S8. qRT-PCR measurement of A20 gene expression relative to B-actin gene (Y-axis) in HepG2 cells treated with Cre adenovirus (Ctrl) and Ad5IκB (IκBα-DN) adenovirus media (X-axis).. 11

Figure S9. Cellular localization of lncTNF in HepG2 cells... 12

Table S1. Plasma and clinical parameters of the MORE study population

	Number of individuals scored	Grade (number of individuals)													
		0	0,5	1	1,5	2	2,5	3	3,5	4	4,5	5	6	7	8
Steatosis (0-3)	60	16	0	15	3	18	/	8	/	/	/	/	/	/	/
Fibrosis (0-4)	58	40	0	9	0	5	1	2	1	/	/	/	/	/	/
Ballooning (0-2)	60	32	1	24	0	3	/	0	/	/	/	/	/	/	/
Lobular inflammation (0-3)	60	23	2	23	0	7	/	5	/	/	/	/	/	/	/
NASH grade (0-3)	60	24	0	24	0	8	/	4	/	/	/	/	/	/	/
NASH stage (0-3)	59	39	0	12	0	4	/	4	/	/	/	/	/	/	/
NAS score (0-8)	60	16	0	2	1	7	0	9	2	14	1	3	1	2	2

	Number of individuals scored	No	Yes
Microgranulomas	59	53	6
Large lipogranulomas	59	42	17
Portal inflammation	60	46	14
Acidofilic bodies	60	54	6
Pigmented macrophages	60	52	8
Glucogenated nuclei	57	48	9

	All individuals		Normal		NAFLD		NASH	
Number of individuals	60		16		8		36	
Males/ Females	14/46		3/13		1/7		10/26	
	Mean	SD	Mean	SD	Mean	SD	Mean	SD
Age (years)	44,05	9,21	41,31	8,68	43,00	7,23	45,50	9,72
BMI (kg/m2)	46,42	10,09	42,50	6,27	45,06	7,02	48,46	11,54
Waist/hip_ratio	1,00	0,12	0,96	0,11	1,02	0,12	1,02	0,12
Glucose (mmol/L)	6,49	2,23	5,70	1,27	5,46	0,63	7,10	2,62
Insulin (mU/L)	17,64	9,81	12,94	6,44	21,13	6,58	19,06	11,10
HOMA-IR (mmol/L)	5,00	3,11	3,22	1,59	5,12	1,56	5,84	3,60
HbA1c (mmol/L)	6,44	1,33	6,03	0,85	6,14	0,57	6,72	1,58
Total Cholesterol (mmol/L)	5,10	1,12	4,91	0,82	5,35	0,80	5,13	1,32
HDL (mmol/L)	1,00	0,35	1,13	0,35	0,99	0,22	0,95	0,36
LDL (mmol/L)	3,21	0,99	3,04	0,80	3,39	0,96	3,25	1,10
TG (mmol/L)	2,29	2,23	1,65	0,77	2,12	0,90	2,67	2,84
FFA (mmol/L)	0,77	0,86	0,47	0,24	0,68	0,22	0,96	1,09
ALAT (U/L)	24,78	9,72	19,88	4,87	28,00	8,82	26,32	10,93
ASAT (U/L)	23,40	12,13	13,88	5,16	26,00	11,49	27,26	12,38
CRP (mg/L)	9,82	8,24	7,62	6,62	11,95	12,11	10,29	7,87

Table S2. Primer sequences

Primer Name	5'-3' primer sequence
Bactin For	AGCCTCGCCTTTGCCGA
Bactin Rev	GCGCGGCGATATCATCATC
RP11-91K9.1 (lncTNF) For	CCCTGTGTGCTGGGATTAAA
RP11-91K9.1 (lncTNF) Rev	TCCATATCAAGTGCATCAAGAA
A20 For	CACTGTGTTTCATCGAGTACAG
A20 Rev	GCAGTATCCTTCAAACATGGTG
IKBA For	CTGGGCCAGCTGACACTAG
IKBA Rev	AGTCATCATAGGGCAGCTCG
U3 snoRNA For	AACCCCGAGGAAGAGAGGTA
U3 snoRNA Rev	CACTCCCCAATACGGAGAGA
DANCR For	CGTCTCTTACGTCTGCGGAA
DANCR Rev	TGGCTTGTGCCTGTAGTTGT
MALAT For	GTGCTACACAGAAGTGGATTC
MALAT Rev	CCTCAGTCCTAGCTTCATCA
NEAT For	CCTCCCTTTAACTTATCCATTCAC
NEAT Rev	TCTCTTCCTCCACCATTACCA
DANCR For	AGGAGTTCGTCTCTTACGTCT
DANCR Rev	TGAAATACCAGCAACAGGACA
OIP5-AS1 For	TGCGAAGAGACCACCAAA
OIP5-AS1 Rev	AGACAACATAGAGACACTTGCC

Figure S1. Induction of inflammation and steatosis in HepG2 cells.

A. qRT-PCR analysis assessing gene expression of A20 and **B**. IκBα genes to confirm successful TNFα treatment. **C. D**. Microscopy images to confirm induction of cellular steatosis by performing Oil Red O staining on HepG2 cells (BSA treated cells in **C** and FFA treated ells in **D**). Upper figures represent 20x magnification and lower 40x magnification. **E**. Oil Red O extraction from lipid droplets of BSA (control) and FFA treated cells, measured by OD assessment on ELISA plate reader at 500 nm.

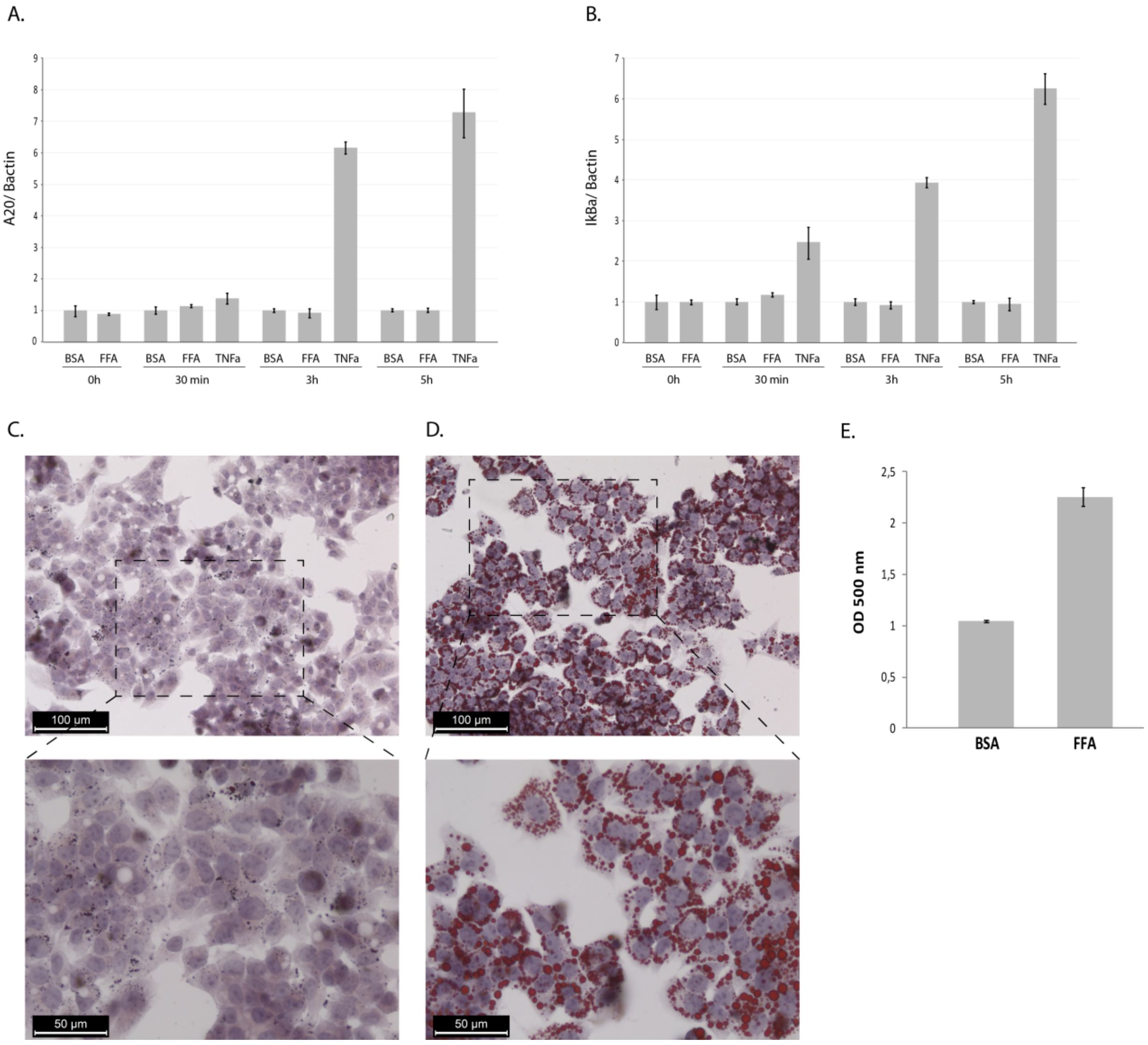

Figure S2. RNA sequencing reads distribution and PC analysis.

A. Distribution of total number of mapped sequencing reads from all 33 samples. **B**. Principal component analysis (PC1 on X-axis and PC2 on Y-axis) on all expressed genes; **C**. on lncNAs alone and **D**. on protein coding gene alone.

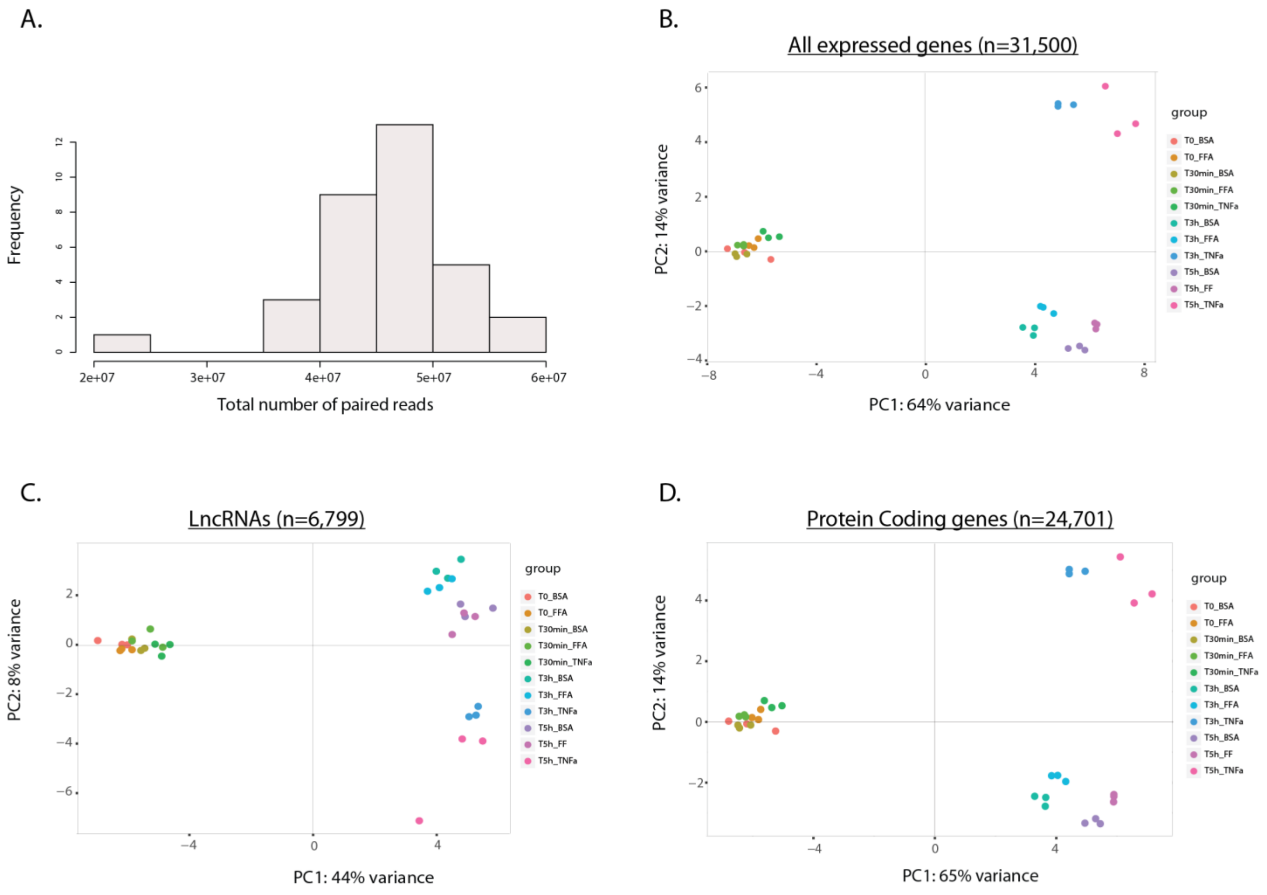

Figure S3. Normalized expression levels of known NASH dis-regulated genes in the HepG2 dataset. X-axis represents normalized gene expression level for the indicated gene and Y-axis represents duration (in hours) for the stimulation/ treatment condition of HepG2 cells: BSA in red line, FFA in green line and FFA+TNFα in blue line.

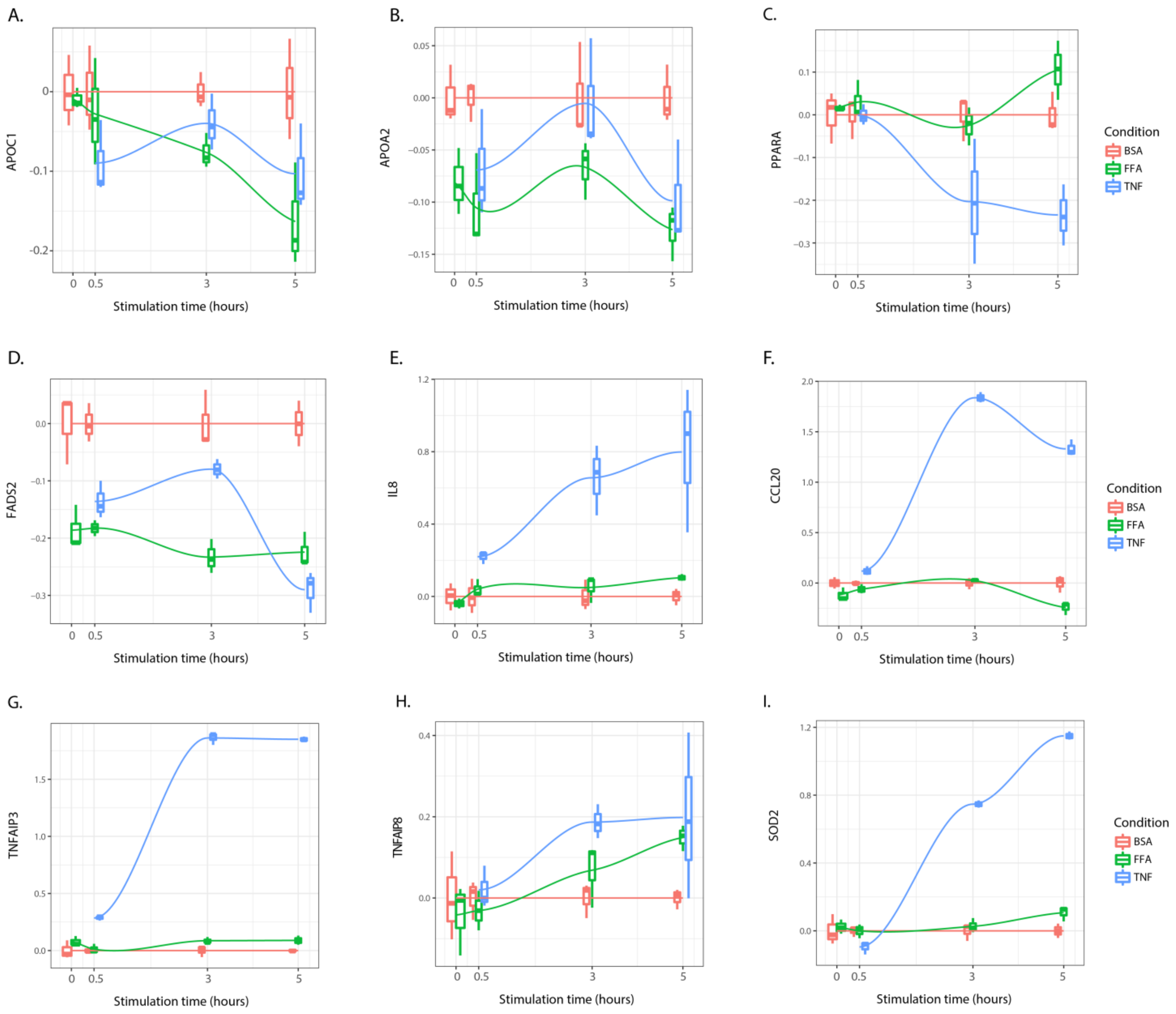

Figure S4. Genes correlated with NAFLD phenotypes in human liver samples.

A. Gene expression heatmap of NAFLD-associated genes (n=3,960) at FDR<0.1. **B.** Bar plot represetnign pathway-enrichment analysis on genes negatively associated with NAFLD/NASH. **C.** Bar plot representing pathway-enrichment analysis on genes positively associated with NAFLD/NASH. Enrichment P-values are presented on the X-axis, pathways on the Y-axis. GO term, Gene Ontology term.

A.

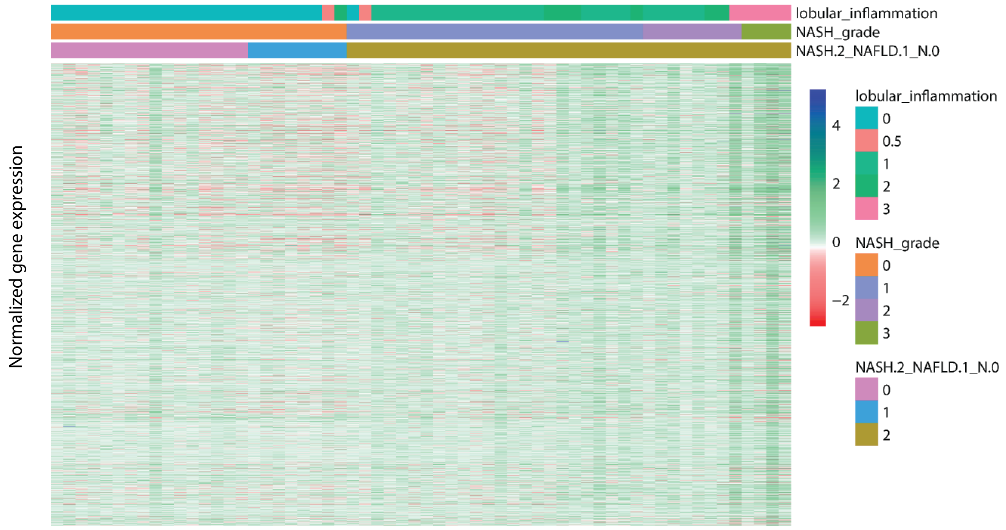

B.

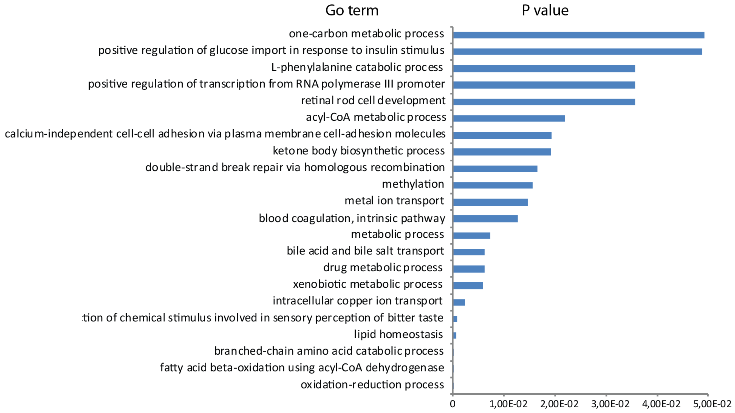

C.

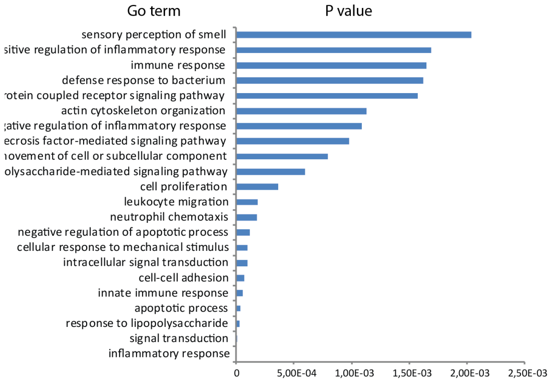

Figure S5. 18 lncRNAs which showed consistent expression patterns in challenged HepG2 cells and in human liver datasets.

A heatmap representing the fold change of each lncRNA gene (Y-axis) relative to the BSA (ctrl) or FFA treatment conditions (X-axis).

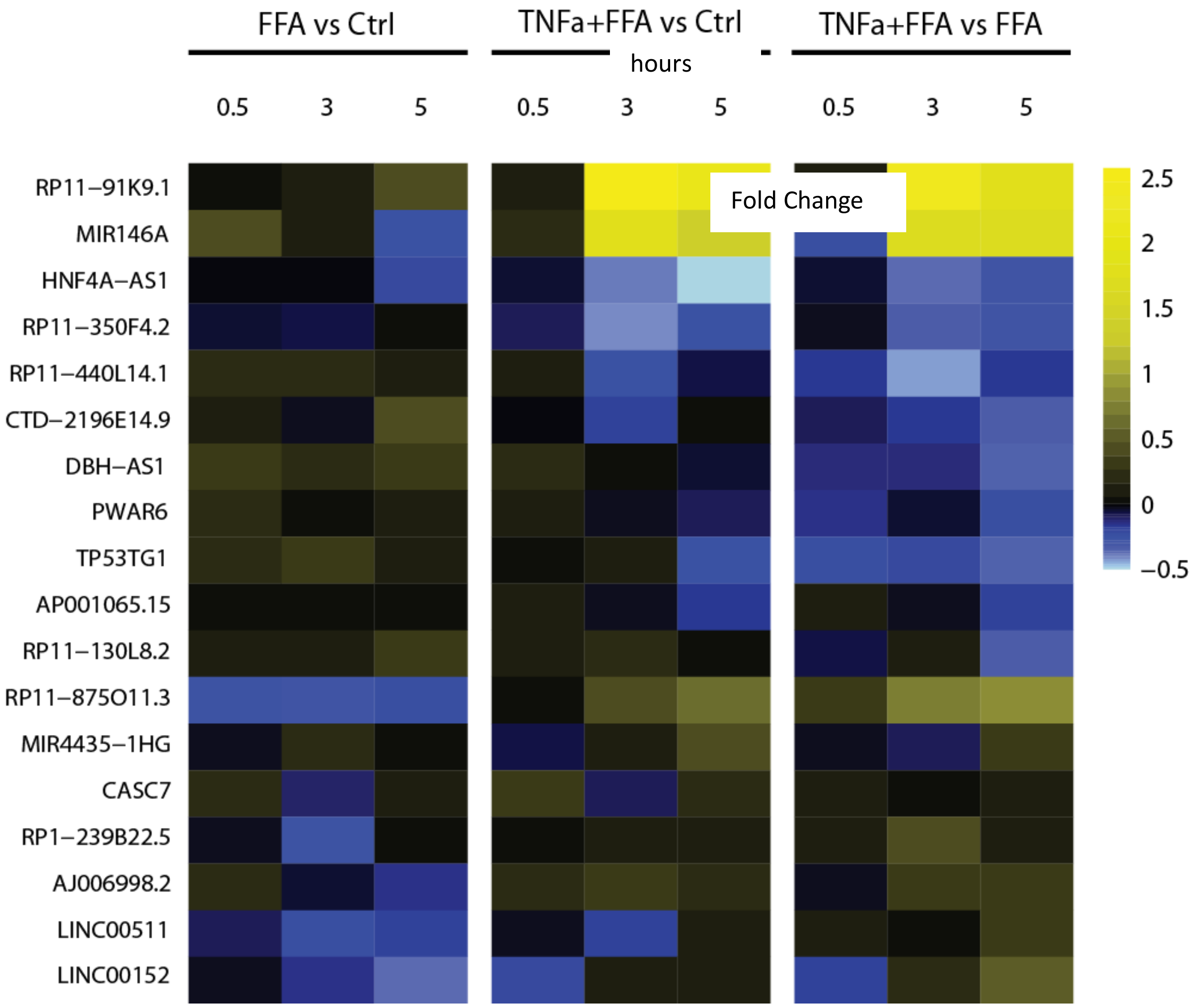

Figure S6. Exprssion levels of lncTNF in publicly available RNAseq datasets (European Nucleotide Archive).

A. Normalized expression levels of lncTNF (Y-axis) across 30 different tissues (X-axis). **B**. Normalized expression levels of lncTNF (Y-axis) across 66 different cell lines (X-axis).

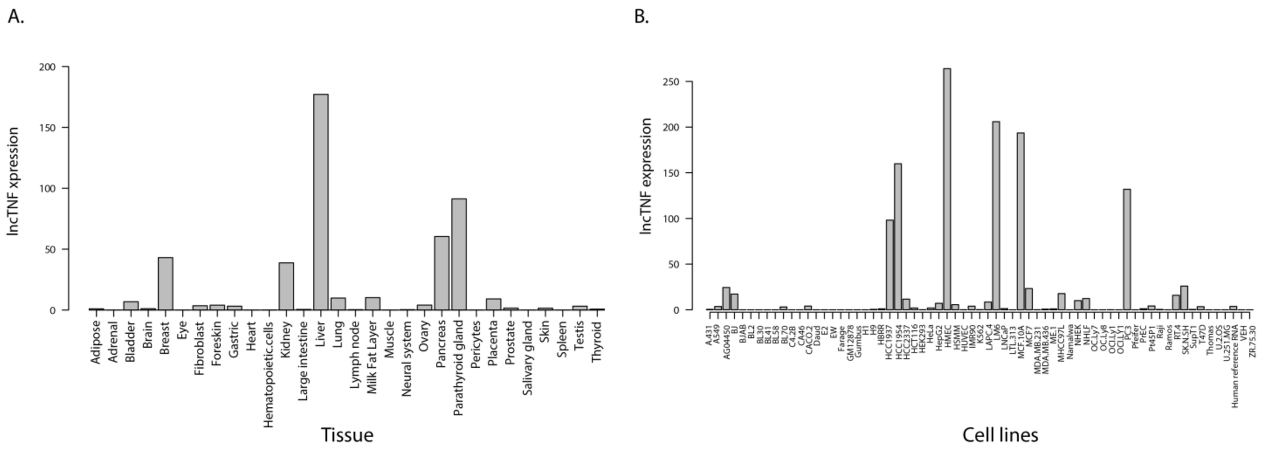

Figure S7. qRT-PCR expression of lncTNF upon different cytokine treatments and among different cell lines.

A. Expression of lncTNF relative to B-actin (Y-axis) in HepG2 cells untreated (0h) or treated with TNFα for 30min-5h time intervals (X-axis). **B.** Expression of lncTNF relative to B-actin in HepG2, Hek293T and THP1 cells in untreated (0h) or TNFα treated (5h) cells. **C**. Expression of lncTNF relative to B-actin in HepG2 cells untreated (0h) or treated with IL1β for 3 and 5h.

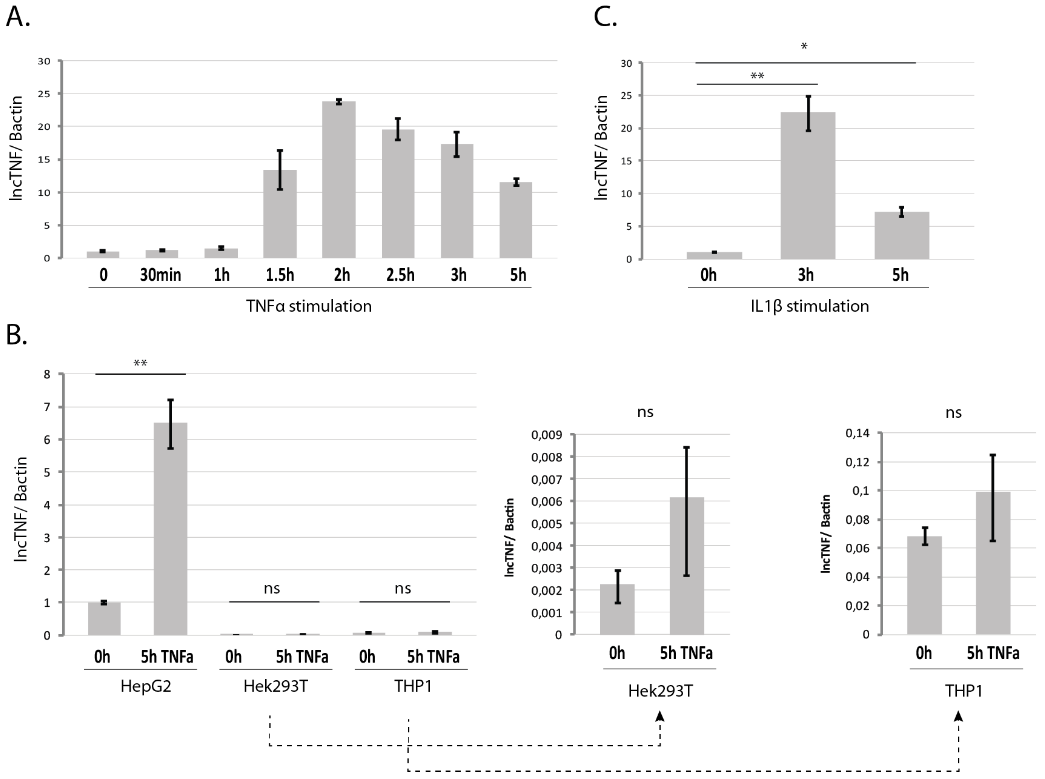

Figure S8. qRT-PCR measurement of A20 gene expression relative to B-actin gene (Y-axis) in HepG2 cells treated with Cre adenovirus (Ctrl) and Ad5IκB (IκBα-DN) adenovirus media (X-axis).

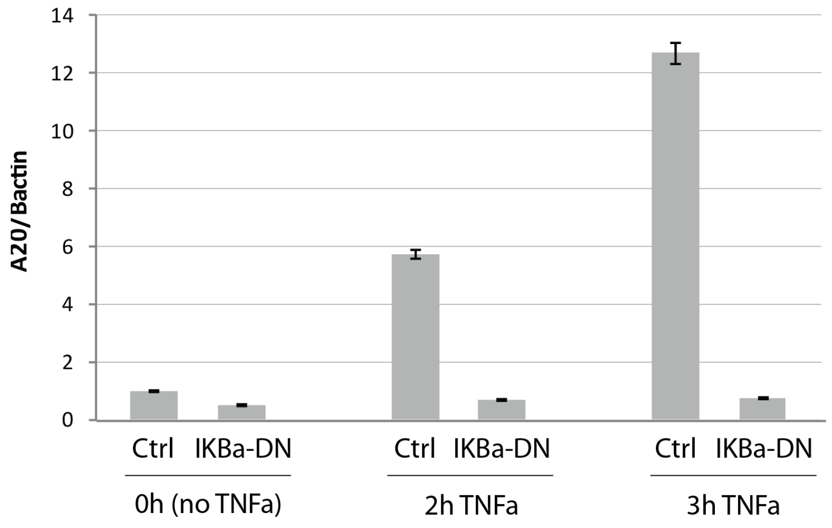

Figure S9. Cellular localization of lncTNF in HepG2 cells.

The localization was assessed by measuring nuclear/ cellular expression ratio of lncTNF. DANCER and OIPS-AS1 were used as controls for lncRNAs expresssed in the cytoplasm, whereas MALAT1 and NEAT1 as controls for lncRNAs expressed in the nucleus.

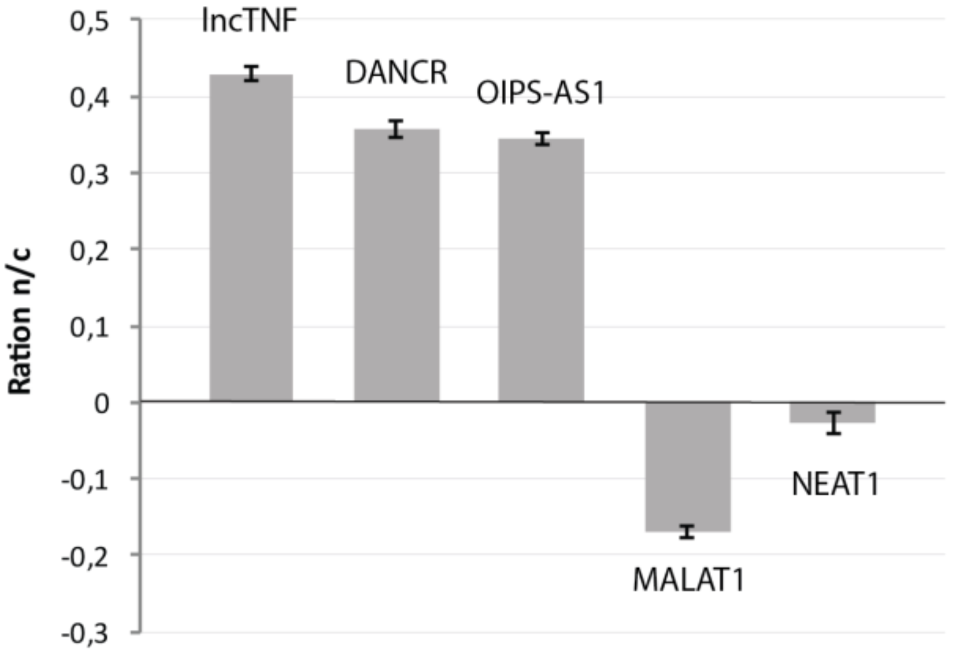

Supplement: Supplementary file 1 [file cells-10-01883-s001.zip › cells-1277694-SI.pdf]
